# Morphology and Function of the Lamb Ileum following Preterm Birth

**DOI:** 10.3389/fped.2018.00008

**Published:** 2018-01-29

**Authors:** Tracey J. Flores, Vivian B. Nguyen, Robert E. Widdop, Megan R. Sutherland, Graeme R. Polglase, Helen E. Abud, Mary Jane Black

**Affiliations:** ^1^The Department of Anatomy and Developmental Biology, Biomedicine Discovery Institute, Monash University, Clayton, VIC, Australia; ^2^Department of Pharmacology, Monash University, Clayton, VIC, Australia; ^3^The Ritchie Centre, Hudson Institute of Medical Research, and the Department of Obstetrics and Gynaecology, Monash University, Clayton, VIC, Australia

**Keywords:** preterm birth, development, gastrointestinal tract, small intestine, ileum, sheep, inflammatory bowel disease

## Abstract

**Background:**

For infants born moderately/late preterm (32–37 weeks of gestation), immaturity of the intestine has the potential to impact both short- and long-term gastrointestinal function. The aim of this study conducted in sheep was to compare the morphology and smooth muscle contractility of the ileum in term and late preterm lambs.

**Materials and methods:**

Lambs delivered preterm (132 days gestation; *n* = 7) or term (147 days gestation; *n* = 9) were milk-fed after birth and euthanased at 2 days of age. A segment of distal ileum was collected for analysis of the length and cellular composition of the villi and crypts, smooth muscle width and contractility, and mRNA expression of the cell markers Ki67, lysozyme, mucin 2, synaptophysin, chromogranin A, olfactomedin 4, axis inhibition protein 2, and leucine-rich repeat-containing G-protein coupled receptor 5 (LGR5).

**Results:**

There was no difference in the proportion of inflammatory, proliferating, apoptotic, enterocyte, or goblet cells between groups, but preterm lambs exhibited a significant upregulation of the stem cell marker LGR5 (*p* = 0.01). Absolute villus height (term: 1,032 ± 147 µm, preterm: 651 ± 52 µm; *p* < 0.0001) and crypt depth (term: 153 ± 11 µm, preterm: 133 ± 17 µm; *p* = 0.01) were significantly shorter in the preterm ileums, with a trend (*p* = 0.06) for a reduction in muscularis externa width. There was no difference between groups in the contractile response to acetylcholine, but peak contractility in response to bradykinin (*p* = 0.02) and angiotensin II (*p* = 0.03) was significantly greater in the preterm lambs.

**Conclusion:**

Findings demonstrate that the crypt-villus units are shorter in the ileum of late preterm offspring, but functionally mature with an equivalent cellular composition and normal contractile response to acetylcholine compared with term offspring. The exaggerated contractility to inflammatory mediators evident in the preterm ileum, however, may be of concern.

## Introduction

Preterm birth, defined as birth occurring before 37 completed weeks of gestation, affects approximately 10% of all live births worldwide, with the majority (~80%) classified as moderately or late preterm (birth between 32 and 37 weeks of gestation) (1, 2). Multiple neonatal complications are associated with preterm birth, including feeding intolerance, intestinal perforations, and necrotizing enterocolitis (NEC) which leads to ischemic necrosis of the gastrointestinal tract (3, 4). NEC predominantly occurs in the most distal aspect of the small intestine, the ileum, and is a multifactorial condition which leads to high-mortality rates (20–30%) in affected infants (3, 4). Under normal conditions, the smooth muscle of the muscularis externa (involved in peristalsis), as well as the epithelium of the intestinal crypt-villus units must function efficiently to achieve adequate digestion and absorption of nutrient intake. However, structural (5, 6) and functional immaturity [dysregulated motility and impaired barrier function (7–9)] of the gastrointestinal tract renders infants born preterm vulnerable to NEC (4, 10).

The feeding protocol of preterm infants is dependent on the age at delivery (11); parenteral feeding is required in extremely preterm infants until the time that the gastrointestinal tract is sufficiently mature to undergo the processes of digestion and absorption. As gestational age at birth increases, enteral feeding is implemented and in infants born only a few weeks early (late preterm) bottle feeding is undertaken (preferably expressed breast milk) (11) as suck–swallow coordination becomes established (12). In these late preterm infants, although intestinal cellular differentiation is sufficient to perform the functions of digestion and absorption (6), they are born during a period of rapid intestinal growth, with a 34% increase in small intestine length observed to occur between 32 and 40 weeks of gestation (5). As such, it is likely that there are still maturational differences in gastrointestinal contractility and in epithelium development of late preterm infants in comparison with infants born at term. These maturational differences can potentially impact the ability of the infant to thrive after birth, and they may also impact long-term gastrointestinal function.

To date, the relative maturity of gastrointestinal contractile function and epithelial development has not been well described in late preterm infants; this is of major clinical importance (13), given that the majority of preterm infants are born during this period (1, 2). Hence, the aim of this study was to compare the contractility of the smooth muscle and structural morphology of the intestinal epithelium of the ileum in term and late preterm offspring. To address this aim, we have used a well-established model of moderate/late preterm birth in sheep, equivalent to birth at approximately 34 weeks of gestation (14, 15). Similar to human infants born late preterm, the preterm lambs are sufficiently mature at birth that they do not require assisted ventilation for breathing, and they are bottle fed expressed milk from the mother. Although the overall structure of the gastrointestinal tract of sheep is different to humans, the anatomical structure of the small intestine closely resembles that of the human making it an appropriate model to examine the effects of preterm birth on the ileum (16–18).

## Materials and Methods

### Animal Studies

All animal experiments were approved by the Monash Animal Research Platform Ethics Committee and complied with the National Health and Medical Research Council’s Code of Practice for the care and handling of animals for scientific purposes.

Time-mated pregnant ewes (Border Leicester × White Suffolk) carrying single fetuses were randomly assigned to give birth at either full term (147 days of gestation; *n* = 9) or preterm (132 days of gestation, ~0.9 of term; *n* = 8). Vaginal delivery was induced in both groups by intravenous administration of Epostane (Sanofi-Synthlabo; Winthrop, UK), an inhibitor of progesterone synthesis, 24 h prior to the expected date of delivery. The ewes allocated to deliver preterm were also administered two intramuscular doses of betamethasone (11.4 mg/dose Celestone Soluspan; Schering-Plough, Baulkham Hills, Australia). The first dose was administered at 130 days of gestation and the second at 131 days of gestation. The dose of betamethasone administered to the ewes was the same as that routinely administered to women at risk of preterm delivery (19).

A detailed description of the animal model has been previously published (14). In the initial period after birth, preterm lambs were bottle fed 80 mL/kg of milk expressed from the ewe, until they were able to independently stand and suckle. Term lambs were allowed to suckle from the ewe immediately following birth. All lambs were humanely killed at 2 days after birth. At necropsy, an approximately 30 cm long segment of distal ileum was removed from the abdomen of each lamb, immediately flushed with 0.9% (w/v) saline solution, and cleaned of connective tissue. As described in Section “Results”, the ileum of one preterm lamb appeared necrotic and this lamb was therefore excluded from all analyses.

### Tissue Collection and Preparation

Initially, the ileum was divided into two separate segments: one segment was cut and rolled in the longitudinal axis, pinned, and fixed in 4% formaldehyde for subsequent histological and immunohistochemical staining (control *n* = 9, preterm *n* = 7), and another segment was collected fresh for organ bath contractility analyses (control *n* = 9, preterm *n* = 4); problems with sample viability (no contractile response) reduced the number of animals that could be analyzed in the preterm group. In subsequent animals, an additional segment of ileum was collected and snap frozen for later mRNA extraction and quantitative polymerase chain reaction (qPCR) analysis (control *n* = 4, preterm *n* = 5).

### Intestinal Smooth Muscle Contractility Analyses

Ileum segments of 1–2 cm in length, were mounted in an organ bath containing Krebs bicarbonate solution (pH 7.4), maintained at 37°C and continuously bubbled with carbogen as previously described (20, 21). Isometric tension was measured through an isometric force transducer (FT-03, Grass Instruments; West Warwick, RI, USA), and data recorded with LabChart (v3.0, AD Instruments; Bella Vista, Australia). The ileum segments were pre-contracted with a high-concentration potassium solution (KPSS: 9.223 g KCl, 0.289 g MgSO_4_.7H_2_O, 2.1 g NaHCO_3_, 0.161 g KH_2_PO_4_, 0.010 g ethylenediaminetetraacetic acid (EDTA), 1.091 g C_6_H_12_O_6_, and 500 µL of CaCl_2_ in 1 L of dH_2_O). Following the maximal contractile response, the organ bath was replaced with fresh Krebs bicarbonate solution, and the tissues allowed to equilibrate. Increasing discrete concentrations of acetylcholine, bradykinin, histamine, and angiotensin II were added to the bath for 2-min intervals (one drug per bath; two pieces of tissue per drug were tested and averaged for each animal). After each 2-min interval, the bath was washed out with fresh Krebs bicarbonate solution, and tissues were left to equilibrate before adding the subsequent concentration. The maximum contractile response at each concentration of acetylcholine, bradykinin, histamine, and angiotensin II was recorded, and analyzed relative to the maximal KPSS response for that tissue.

### Histological Staining for the Detection of Enterocytes and Goblet Cells

Fixed ileum segments were embedded in paraffin and sectioned at 5 µm. Enterocytes, absorptive cells of the intestinal epithelium, were detected by staining for alkaline phosphatase. Sections were dewaxed and incubated in NTMT buffer (pH 9.5) for 10 min, followed by staining using a BCIP/NBT kit (5-Bromo-4-Chloro-Indolyl-Phosphatase/Nitroblue Tetrazolium salt, Invitrogen; Paisley, UK) for 15 min at room temperature. Nuclear fast red was used as a counterstain. Goblet (mucous-secreting) cells were detected using periodic acid Schiff (PAS) stain, and hematoxylin and eosin staining was also performed to assess intestinal morphology.

### Immunohistochemistry for the Detection of Cellular Proliferation, Apoptosis, Inflammatory Cells, and Enteroendocrine Cells

Dewaxed 5 µm paraffin sections underwent heat-induced antigen retrieval in 10 mM sodium citrate buffer (pH 6) for 10 min in a pressure cooker. Sections were then blocked with 0.5% H_2_O_2_ for 30 min followed by CAS Block (Invitrogen; Frederick, MD, USA) for 1 h. Each of the slides were incubated with a primary antibody overnight at 4°C. The primary antibodies used were Ki67, a marker of cellular proliferation (mouse monoclonal 1:100, M7240, Dako; Glostrup, Denmark), cleaved caspase 3, an apoptotic cell marker (recombinant rabbit monoclonal 1:250, 9,661 L, Cell Signaling Technology; Danvers, MA, USA), and synaptophysin (SYP), a marker of enteroendocrine cells (mouse monoclonal 1:500, Dako). Slides were washed and incubated for 30 min at room temperature with a secondary antibody; for Ki67: Envision + Dual Link anti-mouse horse radish peroxidase (HRP) conjugate (ready to use, K4061, Dako), for cleaved caspase 3: goat anti-rabbit HRP conjugate (1:200, Invitrogen; Eugene, OR, USA), and for SYP: goat anti-mouse HRP conjugate (1:200; Invitrogen). Visualization of the immunohistochemical reactions was achieved by incubating slides with 3, 3′-diaminobenzidine in chromogen solution (DAB; K3468, Invitrogen) for 5–10 s for slides analyzed for apoptotic cells, or 5–6 min for slides to be analyzed for proliferating and enteroendocrine cells. Sections were counterstained with hematoxylin. Immunohistochemical staining of Paneth cells was also attempted using anti-lysosyme antibodies, but no specific staining could be visualized in sheep tissue.

Immunohistochemical staining for inflammatory cells using a CD45 (common leukocyte antigen) antibody was performed using a DAKO Autostainer Plus staining system, on sections initially subjected to heat-induced antigen retrieval in sodium citrate buffer. Briefly, sections were incubated with anti-sheep CD45 antibody (1:500; mouse monoclonal MCA2220GA, BioRad) for 1 h, followed by an anti-mouse Dako EnVision + System HRP Labeled Polymer secondary antibody (K4001, Dako) for 1 h. DAB (10 min) was applied to visualize the immunohistochemical reaction, and slides were counterstained with hematoxylin.

### Imaging and Assessment of Villus Height, Crypt Depth, Muscularis Externa Width, and Cell Number

**Figure 1:**
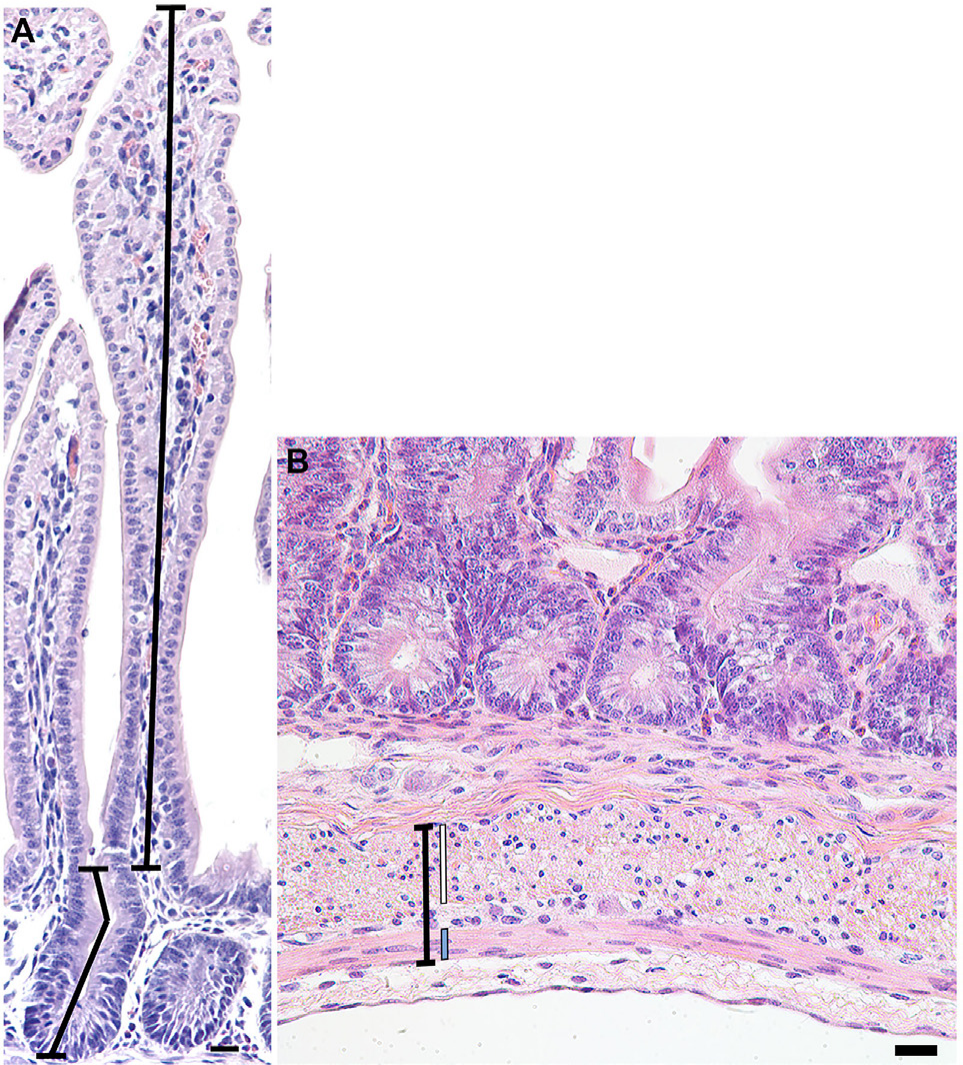
Representative photomicrograph of a crypt-villus unit from a preterm lamb ileum **(A)** stained with hematoxylin and eosin. Crypt depth (lower-left line) was measured from the base of the crypt to the level of the crypt-villus junction, following any curves in the crypt lumen. Villus height (upper-right line) was measured from the crypt-villus junction to the tip of the villus. **(B)** Representative photomicrograph of the muscularis externa (capped black line) from a preterm lamb ileum, stained with hematoxylin and eosin. The inner circular (white line) and outer longitudinal (blue line) smooth muscle layers of the muscularis externa are situated on either side of the myenteric plexus of Auerbach. Scale bars = 20 µm.

Images of 20 randomly selected well-orientated and complete crypt-villus units were captured per animal for each of the stains, using an Axioskop2 plus microscope (Carl Zeiss; Göttingen, Germany) fitted with an Axio Cam MRc5 digital camera (Carl Zeiss). The lens used was relative to the size of the villus, either 10× or 20×. Images of larger villi were captured using a slide autoscanner (Mirax Midi, Carl Zeiss). In hematoxylin and eosin-stained sections, Image Pro-Plus software (v 6.2 Media Cybernetics; Rockville, MD, USA) was used to measure the length of 20 villi and crypts for each lamb, as described in Figure 1A, with average villus height and crypt depth then calculated per lamb.

In 20 randomly selected images of the muscularis externa (Figure 1B), taken using a 40× lens, the width of the inner circular and outer longitudinal smooth muscle layers, as well as the total muscularis externa width (including the myenteric plexus of Auerbach), were measured in two places (40 measurements averaged per lamb) using Image Pro-Plus software (v 6.2 Media Cybernetics; Rockville, MD, USA).

In immunostained sections, the number of caspase 3 + apoptotic cells, and SYP + enteroendocrine cells were assessed in 20 crypt-villus units from the same region of the ileum per animal. The number of Ki67 + proliferating cells per crypt was also assessed. For each stain, the number of positively stained (brown) cells and the number of negatively stained cells were counted, and the average percentage of positively stained cells per crypt-villus or crypt was then calculated for each lamb.

The assessment of enterocyte and goblet cell number was performed in the same way. Goblet cells were identified by a vibrant pink/magenta color (PAS stain), and enterocytes were identified by a blue-stained cellular membrane (BCIP/NBT stain). As goblet cells may also be positive for the BCIP/NBT stain, the number of enterocytes counted was adjusted according to the average proportion of goblet cells counted for that animal.

The number of CD45 + (brown-stained) inflammatory cells was counted in 20 randomly selected crypt-villus units, viewed using a 40× lens. Areas adjacent to Peyer’s patches were excluded. For each crypt-villus unit, a 55 µm × 55 µm (3,025 µm^2^) square region of interest was superimposed over a randomly selected area of villus, the crypt base (extending into surrounding mucosa), and the submucosa directly beneath the crypt (extending into the muscularis exerna); the average number of inflammatory cells in each of the three regions was then calculated for each lamb.

### RNA Extraction and qPCR Analysis

Frozen intestinal segments were incubated in phosphate buffered saline (PBS) with 3 mM EDTA and 0.5 M dichlorodiphenyltrichloroethane for 1 h at 4°C and then washed three times in PBS solution. The intestinal segments were vigorously shaken in PBS for approximately 2 min to dissociate the epithelial cells from the underlying mesenchyme. The resulting supernatant was centrifuged, and the epithelial pellets were stored at −70°C.

Intestinal epithelial pellets were homogenized and total RNA extracted using TRIzol (Life Technologies; Carlsbad, CA, USA) and the RNeasy Mini Kit (Qiagen; Frederick, MD, USA). Reverse transcription was completed using the QuantiTect Reverse Transcription Kit (Qiagen) (22). qPCR was performed using Platinum Taq polymerase (Invitrogen) and SYBR Green (Thermo Fisher; Foster City, CA, USA), with the Mx3000p qPCR system (Stratagene; La Jolla, CA, USA). Amplification conditions were: 95°C for 15 s, 55°C for 30 s, and 72°C for 45 s, and samples were analyzed in triplicate. The resulting curves were analyzed using the comparative ΔCT method with β*-*actin as the housekeeping gene. Primers (Table 1) were specific for the following markers in sheep: β*-*actin (*ACTB*; housekeeping gene), KI67 (marker of cellular proliferation), lysozyme (*LYZ*; marker for Paneth cells), mucin 2 (*MUC2*; marker for goblet cells), *SYP* and chromogranin A (*CHGA*) (enteroendocrine cell markers), and the stem cell markers olfactomedin 4 (*OLFM4*), axis inhibition protein 2 (*AXIN2*), and leucine-rich repeat-containing G-protein coupled receptor 5 (*LGR5*).

**Table 1:** Primer sequences for intestinal cell markers analyzed by qRT-PCR.

Primer	Sequence
β-actin	Forward: 5′GGCATCCTGACCCTCAAGTA
	Reverse: 5′GGGGTGTTGAAGGTCTCAAA
KI67	Forward: 5′GACTCCTGAGAAGGCTGTGG
	Reverse: 5′TGAACTTGCAGGTGGTTGAG
LYZ	Forward: 5′GCCAGATGGGAAAGCAGTTA
	Reverse: 5′AAAGCGCTGCAGGGTATATG
MUC2	Forward: 5′CAGTACAACAGCCACGCCTA
	Reverse: 5′GTATCGTTCGGTTTCCCAGA
SYP	Forward: 5′ACAAAGCCAAAGGGTCTGTG
	Reverse: 5′AAAATGGCAACACACGATCA
CHGA	Forward: 5′TGAACGGATCCTCTCAATCC
	Reverse: 5′CCTCTTCCATCCCTCTTTC
OLFM4	Forward: 5′CCAGCACTGGTAACATCGTG
	Reverse: 5′TTGCCCTGTGTTTGTGTCAT
AXIN2	Forward: 5′CATGGAAAGCGGATACAGGT
	Reverse: 5′TACGCTACTGTCCGTCATGG
LGR5	Forward: 5′CAGGGCAGTCTGTGTCTTCA
	Reverse: 5′CCTTTTACCTGGCACTGGAA

### Statistical Analysis

Data were analyzed using GraphPad Prism (v 5, Graphpad Software Inc.; La Jolla, CA, USA), and are presented as the mean ± SD. Ileum morphology (villus height, crypt depth, and cell number), and gene expression were compared between the term and preterm groups using unpaired two-tailed Student’s *t* tests. Body weights were analyzed using a repeated measures two-way analysis of variance (ANOVA), with the factors preterm birth (p_PT_), postnatal age (*p*_Age_), and their interaction (*p*_PT × Age_). Contractility data (mean peak contraction, relative to peak KPSS contraction) was analyzed using a two-way ANOVA, with the factors preterm birth (*p*_PT_), concentration of drug (*p*_Dose_), and their interaction (*p*_PT × Dose_). These were then followed by Bonferroni *post hoc* tests. *p*-Values <0.05 were accepted as statistically significant.

## Results

### Body Weights

**Figure 2:**
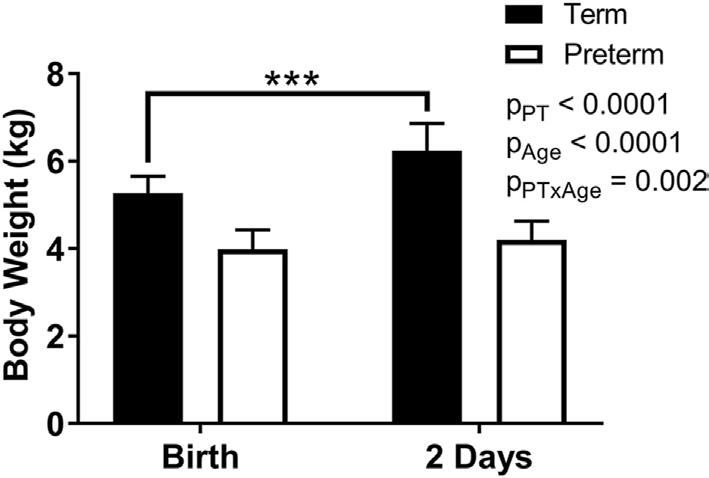
Body weights of term and preterm lambs at birth and at 2 days of age. Data were analyzed using a repeated measures two-way analysis of variance with the factors preterm birth (*p*_PT_), postnatal age (*p*_Age_), and their interaction (*p*_PT × Age_). ***p < 0.0001 according to Bonferroni *post hoc* analysis.

Body weights were significantly lower overall in the preterm lambs compared with the term control lambs (*p* < 0.0001; Figure 2), as well as at birth (*p* < 0.0001) and at postnatal day 2 (*p* < 0.0001). While a significant postnatal increase in body weight was observed in the term animals (birth: 5.3 ± 0.4 kg, 2 days: 6.2 ± 0.6 kg; *p* < 0.0001), there was no significant change in body weight of the preterm lambs over the 2 days (birth: 4.0 ± 0.4 kg, 2 days: 4.2 ± 0.4 kg; *p* = 0.34) (Figure 2).

### Intestinal Smooth Muscle Contractility

**Figure 3:**
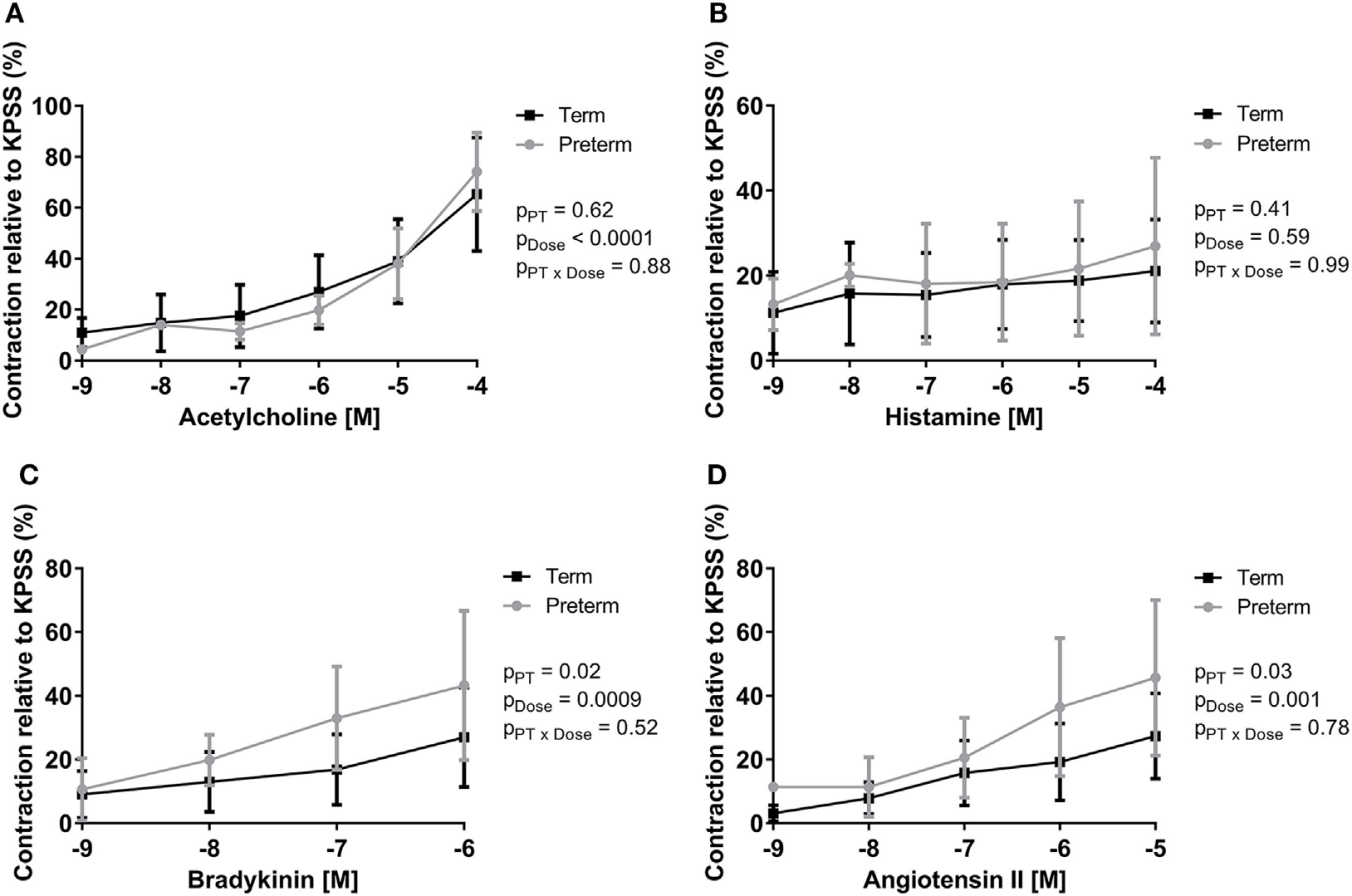
Dose response curves showing peak contractility of preterm and term lamb ileums, relative to KPSS contractility, for acetylcholine **(A)**, histamine **(B)**, bradykinin **(C)**, and angiotensin II **(D)**. Data were analyzed using a two-way analysis of variance with the factors preterm birth (*p*_PT_), drug concentration (*p*_Dose_), and their interaction (*p*_PT × Dose_).

Peak ileal contractile responses to KPSS were not different between groups (term: 1.79 ± 0.62 g, preterm: 1.81 ± 0.62 g; *p* = 0.88). Increasing doses of acetylcholine, bradykinin, and angiotensin II resulted in significant increases in intestinal contraction in all lambs; however, there was no significant dose response to histamine (Figure 3). There was no difference between groups in the peak contractile responses (relative to KPSS) to acetylcholine (Figure 3A) or histamine (Figure 3B). The preterm lambs, however, had significantly greater relative peak contractile responses to bradykinin (*p* = 0.02; Figure 3C) and angiotensin II (*p* = 0.03; Figure 3D) compared with lambs born at term.

### Muscularis Externa Width

**Figure 4:**
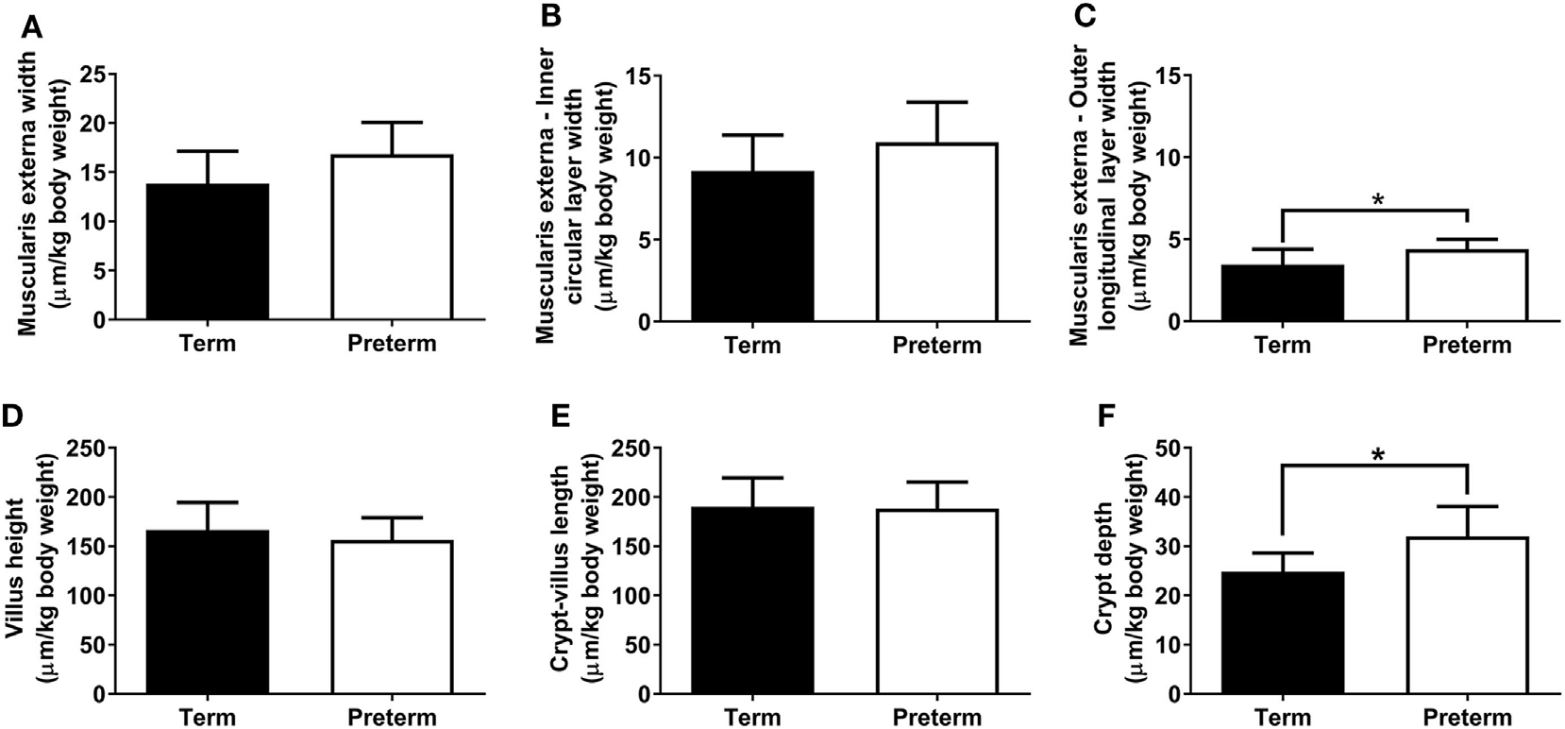
Total muscularis externa width **(A)**, and the width of the inner circular **(B)** and outer longitudinal **(C)** smooth muscle layers, relative to body weight, in term and preterm lambs. Ileum villus height **(D)**, combined crypt-villus length **(E)**, and crypt depth **(F)** relative to body weight in term and preterm lambs. *p < 0.04.

The absolute width of the muscularis externa (term: 85 ± 16 µm, preterm: 70 ± 13 µm; *p* = 0.06) and the inner circular smooth muscle layer (term: 57 ± 11 µm, preterm: 46 ± 10 µm; *p* = 0.059) tended to be smaller in the preterm group, but this did not quite reach statistical significance. There was no significant difference between groups in the absolute width of the outer longitudinal smooth muscle layer (term: 21 ± 5 µm, preterm: 18 ± 2 µm; *p* = 0.14). Relative to body weight, total muscularis externa, and inner circular layer widths were not different between groups (Figures 4A,B); however, the relative width of the outer longitudinal smooth muscle layer was significantly greater in the preterm lambs compared with the lambs born at term (*p* = 0.04; Figure 4C).

### Crypt-Villus Morphology

Absolute villus height (term: 1,032 ± 147 µm, preterm: 651 ± 52 µm; *p* < 0.0001), combined crypt-villus length (term: 1,177 ± 142 µm, preterm: 783 ± 57 µm; *p* < 0.0001), and crypt depth (term: 153 ± 11 µm, preterm: 133 ± 17 µm; *p* = 0.01) were all significantly reduced in the preterm lambs compared with the lambs born at term. When corrected for body weight, there were no significant differences between the term and preterm lambs in ileum villus height or combined crypt-villus length (Figures 4D,E). Relative crypt depth, however, was significantly deeper in the preterm lambs compared with the terms (*p* = 0.01; Figure 4F).

### Proliferation and Apoptosis in the Intestinal Epithelium

**Figure 5:**
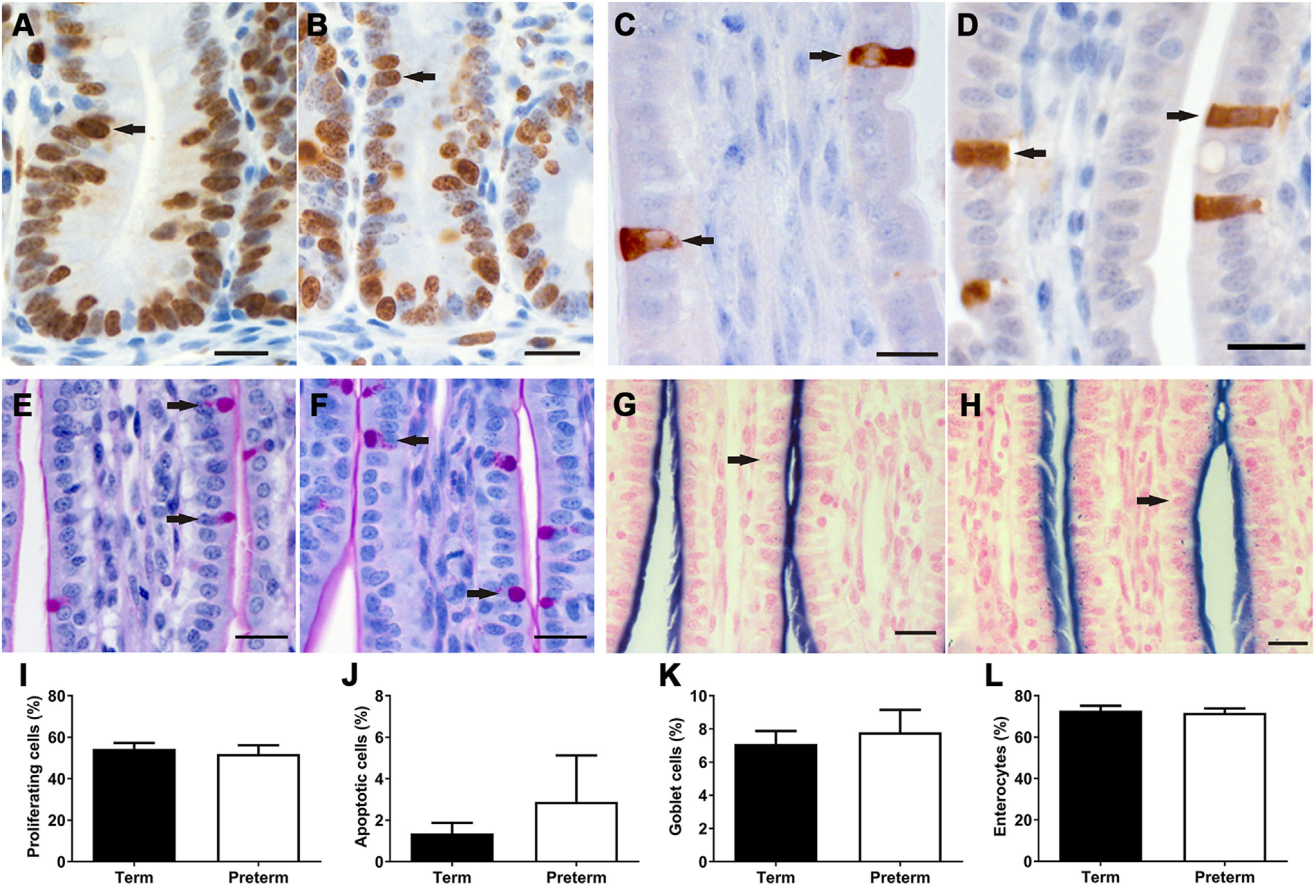
Representative images of Ki67 + proliferating cells **(A,B)** in the crypts and activated caspase 3 + apoptotic cells **(C,D)** in the villi, as indicated by brown staining (arrows), from term **(A,C)** and preterm **(B,D)** lamb ileums. Representative images of periodic acid Schiff-stained villi from term **(E)** and preterm **(F)** lambs, with goblet cells indicated by magenta-colored staining of the cytoplasm (arrows). Representative images of villi from term **(G)** and preterm **(H)** lambs, showing the apical membranes of enterocytes (arrows) colored blue from alkaline phosphatase staining. Scale bars = 10 µm. Graphs show the mean proportion of proliferating cells in the crypts **(I)**, and the proportion of apoptotic **(J)**, goblet **(K)**, and enterocyte **(L)** cells in the crypt-villus units of term and preterm lamb ileums.

In both term and preterm lambs, Ki67 + proliferating cells were found to be restricted to the crypts in the ileum (Figures 5A,B), whereas activated caspase 3 + apoptotic cells were located along the length and tip of the villi (Figures 5C,D). There were no significant differences in the percentage of proliferating (Figure 5I) or apoptotic (Figure 5J) cells between groups.

### Proportion of Intestinal Epithelial Cells

As shown in Figure 5, there were no significant differences in the proportion of goblet cells or enterocytes in the villi of preterm and term lambs. Unfortunately, due to non-specific immunohistochemical staining, the proportion of Paneth cells could not be analyzed in this study. Furthermore, immunohistochemical staining of enteroendocrine cells was only successful in two preterm and seven term lambs; in those ileums, a range of 0–2 enteroendocrine cells were observed per villus.

### Inflammatory Cell Numbers

**Figure 6:**
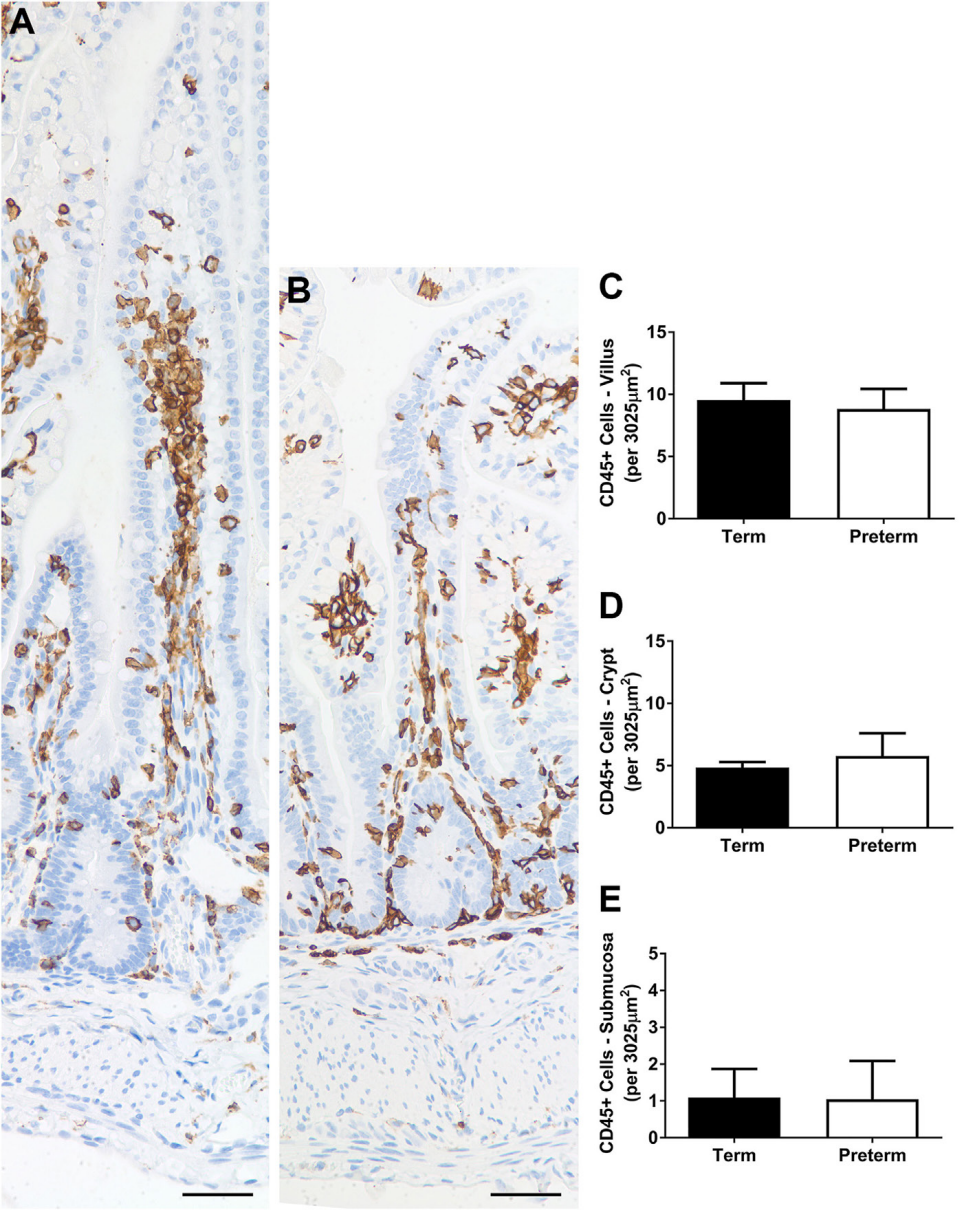
Representative photomicrographs of CD45+ (brown-stained) cells in term **(A)** and preterm **(B)** lamb ileum. Scale bars = 50 µm. Graphs show the mean number of CD45+ cells per unit area (3,025 µm^2^) of villus **(C)**, crypt [and surrounding mucosa; **(D)**], and submucosa **(E)**.

Representative images of CD45 + inflammatory cells in the ileum of term and preterm lambs are shown in Figure 6. The average numbers of inflammatory cells per unit area of villus, crypt, and submucosa were not significantly different between groups (Figures 6C–E). The number of CD45 + cells was most variable in the crypt (and surrounding mucosa) region, ranging from 1 to 12 cells per unit area in the term lambs (an average of 4–5 cells per lamb) and 1 to 17 in the preterm lambs (an average of 3–7 cells per lamb).

### Expression of Intestinal Cell Markers

**Figure 7:**
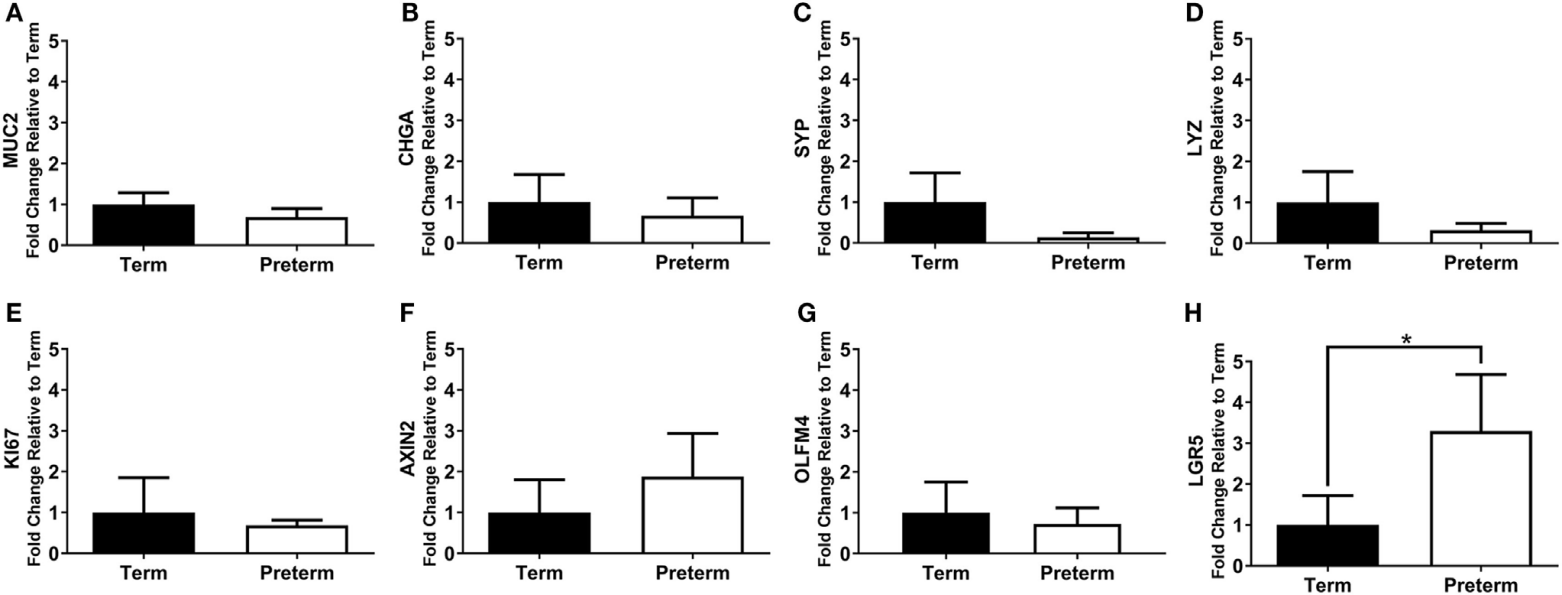
qRT-PCR results, expressed as the fold change in mRNA expression relative to term controls, for the intestinal cell markers *MUC2* **(A)**, *CHGA* **(B)**, *SYP* **(C)**, *LYZ* **(D)**, *KI67* **(E)**, axis inhibition protein 2 (*AXIN2*) **(F)**, olfactomedin 4 (*OLFM4*) **(G)**, and leucine-rich repeat-containing G-protein coupled receptor 5 (*LGR5*) **(H)**. *p = 0.01.

The mRNA expression of intestinal cell markers for goblet cells (*MUC2*), enteroendocrine cells (*CHGA* and *SYP*), Paneth cells (*LYZ*), proliferating cells (*KI67*), and stem cells (*AXIN2, OLFM4*, and *LGR5)* are shown in Figure 7. Expression of the stem cell marker *LGR5* was significantly increased in the preterm ileum compared with the terms (*p* = 0.01; Figure 7H), but there were no statistically significant differences between groups for any other marker.

### Necrosis in One Preterm Ileum

**Figure 8:**
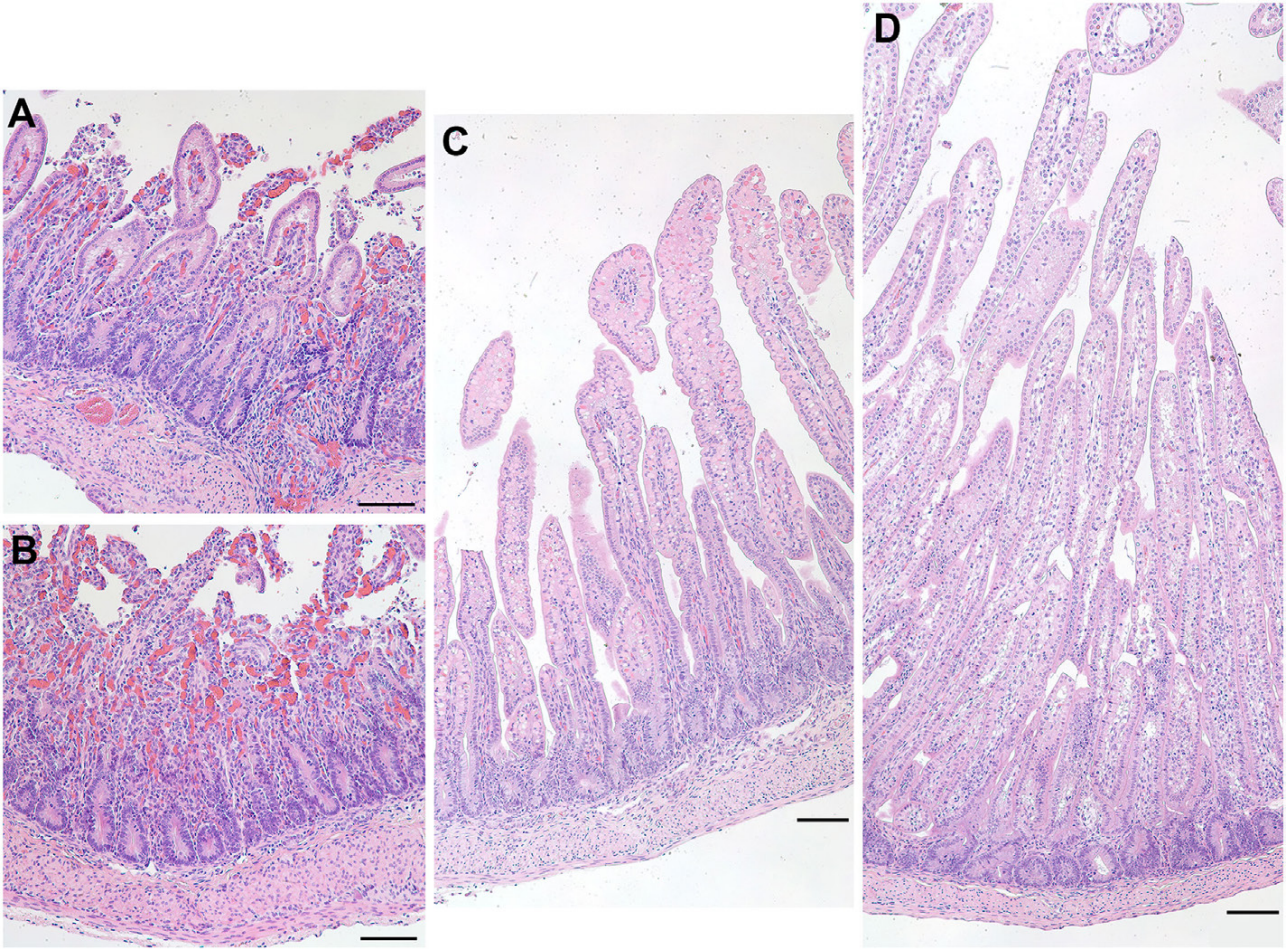
Representative images of hematoxylin and eosin-stained sections of the ileum from a preterm lamb (excluded from analysis) that exhibited epithelial degradation along the villi and cellular debris suggestive of necrotizing enterocolitis **(A,B)**. A normal preterm lamb ileum **(C)** and a normal term lamb ileum **(D)** are shown for comparison. Scale bars = 100 µm.

At the time of necropsy, one preterm lamb appeared to show signs of physical weakness. During harvesting of the ileum, it was clear that the tissue was a darker shade of red and was also very fragile to handle. Upon closer inspection of the ileal epithelium, it was clear that there was degradation as well as a vast amount of cellular debris apparent throughout the tissue (Figure 8). These characteristics are suggestive of NEC.

## Discussion

Although infants that are born late preterm do not usually exhibit overt gastrointestinal dysfunction, the findings of this study clearly demonstrate differences in the contractility, gene expression, and cellular structure of the ileum, with a heightened contractile response to inflammatory mediators.

Overall, there was no evidence to demonstrate impairment of the contractility of the ileum in the late preterm intestinal segments given that the dose response to acetylcholine was not different between the preterm and term groups. Acetylcholine (acting *via* muscarinic receptors) is the normal mediator of intestinal contractility (23). Therefore, given that the functional response to acetylcholine was not different between the intestinal segments from the preterm and term lambs, this suggests that the muscarinic receptors were fully developed and functionally mature in the preterm lambs. Hence, by 132 days gestation (equivalent to ~34 weeks in humans) the smooth muscle of the lamb ileum appears to have reached equivalent contractile maturity as 2-day-old term lambs. Supporting these findings, in a previous study by Oyachi et al. (24) it was shown that the ileal contractile response to bethanechol (another cholinergic agent) is similar between fetuses at 130-day gestation and term-born lambs at 1 week of age; age-dependent differences in the expression and sensitivity of the muscarinic receptor subtypes were also observed. Previous studies have also demonstrated that human preterm infants at 34 weeks of gestation have largely mature intestinal motility, with regulated patterns of migrating motor complexes observed at this age (25, 26).

Importantly, however, the increased dose-dependent contractile response to bradykinin and angiotensin II in the preterm lamb ileums is indicative of a heightened vulnerability to intestinal inflammation (27, 28). Interestingly, this increased contractility in the preterm ileum occurred in the context of a significantly greater width of the outer longitudinal layer of the muscularis externa, relative to body weight, than the term lambs. Bradykinin is a local inflammatory mediator (27), and in the term ileum there was a small dose-dependent increase in the contractility to bradykinin. In contrast, however, this response was markedly increased in the preterm lambs. Significantly increased contractility of the preterm ileum was similarly observed in response to angiotensin II, a major component of the renin–angiotensin system (RAS) with roles in absorption, secretion, and direct and indirect stimulation of smooth muscle contraction in the small intestine (28). It is possible that age-dependent differences in the expression and sensitivity of the angiotensin and bradykinin receptors in the ileum [as has been shown for the muscarinic receptors (24)] may underlie these contractile differences between groups, and further studies would be required to confirm this. There is now a growing body of evidence from experimental and clinical studies in adults linking activation of the local gastrointestinal RAS (28–30) and kinin pathways (31–33) with the pathophysiology of inflammatory bowel disease (ulcerative colitis and Crohn’s disease). Our novel findings of a much greater dose-dependent response to angiotensin II and bradykinin in the ileum of preterm lambs compared with term lambs, suggests that these pathways may also play a role in the pathophysiology of gastrointestinal inflammatory disorders associated with preterm birth, and this warrants further investigation. Indeed, one study has identified preterm birth as an independent risk factor for the development of inflammatory bowel disease later in life (34).

The exaggerated contractility to both bradykinin and angiotensin II suggests that the preterm ileum is hyper-reactive to inflammatory contractile stimuli. This is important, given that the significant upregulation of LGR5 in the preterm ileum is suggestive of a regenerative response which could occur in response to underlying intestinal inflammation following late preterm birth. LGR5 functions as a receptor for R-Spondin which can enhance WNT signaling in intestinal crypt base columnar stem cells that drive homeostatic renewal of the intestinal epithelium (35–37). Studies in fetal gut have also demonstrated the involvement of LGR5 in regulating the differentiation of Paneth (antimicrobial peptide-secreting) cells (38). The upregulation of LGR5 is linked to intestinal inflammation, whereby there is an expansion of the intestinal stem cells in order to replenish the intestinal epithelium (39–41). The number of CD45 positive leukocytes was not significantly different between groups, however, suggesting that the preterm tissue in the majority of lambs had not progressed to an overt inflammatory phenotype. Excessive inflammation of the preterm ileum is a well-recognized feature of NEC, and is thought to occur due to immaturity of the innate immune response (42). It is important to note that in one of the preterm lambs there was pathological evidence of villi degradation, suggesting that this lamb may have subsequently developed NEC in the neonatal period.

A limitation of this study was that not all analyses could be conducted in every animal. In particular, for a number of preterm ileums a contractile response was not elicited, which prevented any functional analyses. The reasons for this are unknown, but there is a possibility that our contractility findings are biased toward the healthiest of the preterm animals. However, we did structurally assess the ileum morphology of all animals, and except for the one preterm lamb excluded from analysis due to villus degradation, no other preterm lambs exhibited any signs of structural abnormalities. The lower sample size for these contractility analyses, as well as for RT-PCR (due to commencement of these analyses later in the project), would potentially, however, have limited the power to detect differences between groups.

In accordance with our findings of contractile maturity being reached by 132 days of gestation in our late preterm lambs, there were no major differences detected in the epithelial cell population, suggesting that the epithelial lining of the ileum had also reached functional maturity by 132 days of gestation. There was no difference in the proportion of enterocytes or goblet cells in the villus-crypt units in the ileum of late preterm lambs when compared with term lambs, which was directly assessed in histological sections. Likewise, there were no differences in the mRNA expression of the epithelial cell proteins associated with goblet cells (mucin), enteroendocrine cells (chromogranin and SYP) and Paneth cells (lysozyme); however, further studies are required to confirm this at the protein level. Absolute villus height and crypt depth were significantly lower in the preterm lambs in comparison with the terms, which corresponds with the previous findings of Trahair et al. (18) showing that both measures in the fetal sheep small intestine increase with increasing age. Similarly, muscularis externa width tended to be lower in the preterm lambs than the terms. Relative to body weight, however, there was a significant increase in crypt depth, and the width of the outer longitudinal smooth muscle layer, in the preterm lambs compared with the terms, which reflects the fairly narrow difference in crypt depth/smooth muscle width (but large difference in body weight) between groups. The number of proliferating cells was far greater than the number of apoptotic cells in the crypt-villus units of both the preterm and term lambs, indicating a highly proliferative state of growth. Given that there was no difference in the proportion of proliferating or apoptotic cells in the intestinal crypt-villus units of the preterm and term lambs, our findings imply that the rate of epithelial cell turnover is likely similar between groups; time-course studies, whereby epithelial cells are labeled (such as with BrdU) and followed over a number of days, would be required to confirm this.

In conclusion, the findings of this study demonstrate, using an ovine model, that the intestinal smooth muscle and villus epithelium of the ileum is functionally mature in late preterm offspring; hence, the ileum appears to be functionally prepared for postnatal life. The exaggerated contractility to inflammatory mediators, however, is of concern given that there was also evidence of upregulation of a stem cell marker linked to intestinal stress and inflammation. Importantly, if this exaggerated contractility to inflammatory mediators persists into postnatal life it may be a plausible explanation for the link between preterm birth and inflammatory bowel disease (34). Future studies that extend further into the neonatal period and into childhood will help to elucidate whether late preterm birth subsequently leads to long-term deleterious impacts on gastrointestinal function.

## Ethics Statement

All animal experiments were approved by the Monash Animal Research Platform Ethics Committee and complied with the National Health and Medical Research Council’s Code of Practice for the care and handling of animals for scientific purposes.

## Author Contributions

All authors made substantial contributions to the conception or design of the work, or the acquisition, analysis, or interpretation of data for the work. All authors were involved in drafting the work or revising it critically for important intellectual content. All authors gave final approval of the version to be published, and agree to be accountable for all aspects of the work in ensuring that questions related to the accuracy or integrity of any part of the work are appropriately investigated and resolved.

## Conflict of Interest Statement

This research was conducted in the absence of any commercial or financial relationships that could be construed as a potential conflict of interest.
